# Comparison of Intravenous Midazolam Drip with Intermittent Intravenous Diazepam in the Treatment of Refractory Serial Seizures in Children

**Published:** 2012

**Authors:** Afshin FAYYAZI, Parvaneh KARIMZADEH, Saadat TORABIAN, Somayeh DAMADI, Ali Khajeh

**Affiliations:** 1 Assistant Professor of Pediatric Neurology, Hamedan University ofMedical Sciences, Hamedan, Iran; 2 Professor of Pediatric Neurology, Pediatric Neurology Research Center, Shahid Beheshti University of Medical Sciences (SBMU), Tehran, Iran; 3 Professor of Pediatric Neurology, Department of Pediatric Neurology, Mofid Children Hosptial, Shahid Beheshti University of Medical Sciences (SBMU), Tehran, Iran; 4 Assistant Professor of Community Medicine, Hamedan University of Medical Sciences, Hamedan, Iran; 5 General Practitioner, Hamedan, Iran; 6 Assistant Professor of Pediatric Neurology. Children & adolescent health Research center, Zahedan University of Medical Sciences, Zahedan, Iran

**Keywords:** Seizures, Refractory, Children, Midazolam, Diazepam

## Abstract

**Objective:**

Serial seizures occur commonly in inpatient epileptic children. This type of seizure due to its characteristics has a significant impact on the patient’s health.

Untreated serial seizures lead to status epilepticus; therefore, finding a more effective treatment for such patients is essential. This study was performed to compare the outcome of intermittent intravenous diazepam in the pediatric neurology clinic and intravenous midazolam in the pediatric intensive care unit (PICU), in order to introduce an alternative treatment for serail seizures.

**Materials & Methods:**

In this study, 38 inpatient children aged 6 mo-15 years with refractory serial seizures were treated by first line antiepileptic drugs and then randomly treated with either intermittent intravenous diazepam in the neurology ward or intravenous midazolam in PICU.

**Results:**

Fourteen (70%) diazepam group patients and 13 (72.2%) midazolam group patients had good response to treatment, there was no significant difference between the two groups. Four midazolam group patients and two diazepam group patients needed mechanical ventilation and were intubated during treatment, with no significant difference between the two groups. Durations of mechanical ventilation and PICU and hospital stay were not significantly different between the two groups.

**Conclusion:**

Intermittent intravenous diazepam is an effective alternative therapy for midazolam drip in the treatment of serial seizures due to similar therapeutic effects and fewer side effects.

## Introduction

Serial seizures, also known as repetitive or cluster seizures, occur commonly in children with epilepsy (1). At the simplest level, serial seizures have been defined as the number of seizures per unit time with an increased frequency over the patient’s typical seizure (1).

There have been several definitions for serial seizures clinically and statistically according to different studies. In studies with a clinical definition for serial seizures, cluster patterns have been defined differently; including two to four seizures in a short period of ≤48 hours (2); three or more seizures within 24 hours (3, 4); two generalized tonic-clonic or three complex partial seizures in 4 hours (5); and a three-fold or four-fold increase over usual seizure frequency within a three-day period (6, 7). From the statistical point of view, serial seizures are those without random distribution, or with a dependence pattern of interseizure interval (1). Some studies have applied Poisson distribution test to seizure frequency, to evaluate the randomness of seizures (8, 9).

The prevalence of clustering varies widely between studies because there is no definitive clinical definition for serial seizures. Whether clinically or statistically defined, the prevalence of serial seizures ranges from very low up to 60 % (1). 

Various studies have suggested risk factors for serial seizures, such as epilepsy localization, particularly frontal lobe epilepsy; head trauma and a history of intractable epilepsy (1).

Serial seizures although not as life threatening as status epilepticus, have a significant effect on the patient’s health (10) and if left untreated, have been reported to progress into status epilepticus (11, 12) indicating a poor prognosis of epilepsy (1). The socioeconomic effects of serial seizures include missed school days, as well as increased consumption of health care resources. 

In most hospitals, the first approach as the routine approach for refractory serial seizure is admission of the patient in the Pediatric Intensive Care Unit (PICU) and starting intravenous midazolam infusion. Limited PICU services in some remote hospitals were the main drawback of this approach. In addition, infusion of midazolam drip cannot be administered in the pediatric neurology ward. Therefore, treatment of refractory serial seizures sometimes becomes a challenging problem. 

The aim of this study was achievement of a new method for treatment of patients with serial seizures when transfer to the PICU is not possible.

## Materials & Methods

This study included all admitted children with refractory serial seizures in Mofid hospital, which is a referral center for pediatric neurological diseases, aged from 6 months to 15 years, from October 2008 to May 2010.

The diagnostic criteria for refractory serial seizures in this study was defined as four generalized tonic-clonic or complex partial seizures per day with a three-fold increase over usual seizure frequency within a two-day period that received at least two appropriate intravenous, intramuscular or oral antiepileptic drugs with no decrease to less than the pretreatment level in seizure frequency. Occurrence of status epilepticus due to convulsive seizure longer than 30 minutes was considered as exclusion criteria, in addition to critical diseases such as meningitis and intracranial hemorrhage.

Patients were randomly assigned to midazolam or diazepam groups. The patients were transferred to PICU and in the midazolam group, midazolam was administered as an intravenous bolus dose (0.2mg/kg), followed by continuous intravenous infusion (1-10 μg/ kg per min) and in the diazepam group, intravenous diazepam was administered every 3 hours (0.2 mg /kg). 

Decreased seizure frequencies to less than half of pretreatment level or complete seizure disappearance (for at least 48 hours after discontinuation of these drugs) were considered as effective treatment. Decrease in seizure frequency, but not less than the pretreatment level, was considered as partial response and finally no difference was assumed as not effective. 

All children were monitored for the development of side effects of midazolam and diazepam, such as hypotension and respiratory depression. Routine laboratory examinations and EEG were performed in all patients. Brain CT or MRI was performed if needed.

Data including age, sex, seizure history, underlying etiology and outcome were carefully recorded for each patient. We compared the two groups by independent sample t test, Fisher’s exact test or Mann-Whitney U test. A value of P<0.05 was regarded as significant. 

This study has been approved by the ethics committee of Shahid Beheshti University of Medical Sciences, Tehran, Iran.

## Results

Overall, 44 children were eligible for our study. We excluded six children owing to early death before treatment (one patient), transport to other hospitals (one patient) and critical situation other than seizure (four patients).

Finally, 38 patients, 18 boys and 20 girls (range, 6 months to 15 years) were recruited as refractory serial seizures (RSS) in this study, 18 patients in the midazolam and 20 

## Discussion

To the best of our knowledge, no controlled studies have compared the efficacy of intermittent intravenous diazepam with intravenous midazolam drip for the management of serial seizures. We found that intermittent diazepam given intravenously is as safe and effective as midazolam drip given intravenously in the management of refractory serial seizures in children.

Some investigators have reported the efficacy of rectal diazepam in serial seizures (13, 14). Dreifuss et al (13) reported an efficacy of rectal diazepam comparable to that obtained with placebo in serial seizures (P< 0.001).

The safety and efficacy of midazolam has been shown by several clinical studies in the treatment of acute seizures and status epilepticus in adult and children (15-18). Midazolam has also successfully controlled serial seizures in children (19). The current study showed that not only the adverse effects of diazepam were not more than midazolam, but also the duration of admission in PICU in the midazolam group were longer than the diazepam group.

Hayashi et al (18) detected that 20% of the patients with status epilepticus treated with midazolam drip had respiratory suppuration and needed mechanical ventilation. This rate in our study was 22.2%, but our patients were clinically different. In that study, 64.5% of the status epilepticus patients had good responses to midazolam drip compared to the 66.6% rate in our study. 

Early termination of serial seizures is important to prevent adverse consequences and to reduce the risk of development of status epilepticus. 

**Table 1:** Comparative Baseline Characteristics of the Two Groups of Children

	**Midazolam**	**Diazepam**	**P Value**
Chronologic age (mo)	48.78 ± 38.516	71.05 ± 54.401	o.081
Age of onset of first seizure (mo)	19.72 ± 22.932	28.15 ± 43.274	0.274
Gender (Male/Female)	0.80	1.00	0.732
Neurodevelopment disability (%)	61.1	45	0.321
Category of seizure (No. of children)			
Idiopathic epilepsy Cryptogenic epilepsy Symptomatic epilepsy	7 1 10	9 3 8	

**Table 2:** Duration of Mechanical Ventilation and Hospital and PICU Stays

	**Midazolam**	**Diazepam**	**P Value**
Duration of hospital stay (day)	15.83 ± 14.460	11.10 ± 7.779	0.063
Duration of PICU stay (day)	10.89 ± 9.305	3.20 ± 6.305	0.106
Duration of mechanical ventilation	4.61 ± 7.912	1.35 ± 5.174	0.054

**In conclusion,** our study showed that intermittent intravenous diazepam may be considered as an effective alternative therapy for midazolam in the treatment of serial seizures, because of similar therapeutic effects and less side effects.
